# Workplace Challenges and Policy Responses in the Caribbean Nursing Workforce: Insights From Country Chief Nurses

**DOI:** 10.1111/nhs.70320

**Published:** 2026-03-15

**Authors:** Eileen T. Lake, Domenique Villani, Lynne Moronski, Lindsey Lee, Sherif Olanrewaju, Norah Solaiman, Claire Burke Draucker

**Affiliations:** ^1^ Center for Health Outcomes and Policy Research University of Pennsylvania School of Nursing Philadelphia Pennsylvania USA; ^2^ Georgetown University Berkley School of Nursing Washington D.C. USA; ^3^ Indiana University School of Nursing Indianapolis Indiana USA

**Keywords:** Caribbean region, health workforce, nurses, Pan American Health Organization, policy, working conditions

## Abstract

The Caribbean faces significant nursing and midwifery workforce challenges. A 2023 PAHO policy advises Member States to develop and implement strategies to strengthen their health workforce, including “promoting decent work conditions.” Caribbean nurses' work conditions have not been described. The country's chief nurse has responsibility for policy development. The study purpose was to describe the Caribbean country chief nurses' concerns about nurses' work environments, strides made and polices implemented to support nurse work environments and quality care. Chief nurses of 20 Caribbean countries were surveyed. Inductive content analysis was done. Five concerns represented: (1) physical environment; (2) staffing and workload; (3) safety and security; (4) professional development; and (5) nurses' well‐being. Improvements and policies reflected similar themes. The predominant work environment concerns center on inadequate physical environment, nurse staffing and workloads, and inconsistent security. The sporadic improvements and few policies initiated in several countries may warrant coordinated regional effort to achieve broader improvement in working conditions. To optimize patient care and a healthy, safe nursing workforce, policymakers should consider the priorities of government chief nurses to improve working conditions.

## Key Points

- This study employs a qualitative descriptive design to describe government chief nurses' concerns, strides, and policies related to improving nurses' work environments across 20 Caribbean countries in context of major health workforce challenges in the Caribbean region.
- Chief nurses identified five main areas of concern—physical environment, staffing and workload, safety and security, professional development, and nurses' well‐being—with improvements and policy actions largely reflecting these same themes but occurring inconsistently across the region.
- Our findings highlight the need for coordinated regional and international efforts to prioritize chief nurses' concerns and to strengthen nursing work environments.

## 1 Introduction

Improving nurses' work conditions is a key strategy to strengthen the health workforce in the Pan American region (Pan American Health Organization 2023). The Caribbean faces significant nursing workforce shortages compounded by high out‐migration (i.e., leaving the home country) and the COVID‐19 pandemic, threatening the stability and effectiveness of health systems. To address these challenges, the 60th Directing Council of the Pan American Health Organization (PAHO) approved the “Policy on the Health Workforce 2030: Strengthening Human Resources for Health to Achieve Resilient Health Systems” (Pan American Health Organization 2023). This policy aims to guide Member States to strengthen their health workforce, emphasizing “promoting decent work conditions” among five key objectives.

We utilize two related terms: work conditions and work environment, for how to support our human resources to achieve successful health systems. We clarify their meaning from policy guidance and known constructs in the nursing systems literature.

Nurses' work conditions lack a single, precise definition. Authoritative reports provide varying elements. The first State of the World's Nursing report identified adequate remuneration, social protection, fair working conditions, reasonable working hours, occupational safety, non‐monetary incentives, and transparent and merit‐based opportunities for career progression (World Health Organization 2020). A recent PAHO policy brief defined work conditions as working hours, workload, and access to modern medical equipment and facilities (Pan American Health Organization 2024). The State of the World's Nursing 2025 report included wages and pay structures, workplace safety and violence, gender discrimination, and career progression opportunities (World Health Organization 2025b). Given these varying elements, we consider working conditions to encompass working hours/workload, safety, physical environment, and career opportunity.

Similarly, the work environment has various definitions. Lake (2007) described a professional practice environment as one that “supports nurses to function at the highest scope of clinical practice, to work effectively in an interdisciplinary team of caregivers, and to mobilize resources quickly.” A systematic review of 54 studies concluded that the “work environment is a broad construct and a collective term signifying healthcare organizational culture and patient care environments.” (Wei et al. 2018, 297). In this study, we treat the work environment as a component of nurses' broader work conditions. Considerable international evidence links nurse work environments to retention, satisfaction, quality, safety, and patient outcomes (Lake et al. 2019; Wei et al. 2018).

The State of the World's Nursing 2025 report documents global unsafe staffing, unequal pay, inadequate mental health protections, and weak regulation of working conditions that accelerate burnout and international migration (World Health Organization 2025b). PAHO projects a regional shortage of at least 600 000 health professionals by 2030, with nurses and midwives accounting for the largest gap, an estimated 1.57 million shortfall, because 93.9% of countries fall below minimum workforce density thresholds (Pan American Health Organization 2023). Caribbean nations experience some of the world's highest nurse emigration rates, with over half of Caribbean‐trained nurses working in high‐income Organization for Economic Co‐operation and Development (OECD) countries. 92% of Caribbean health professionals report they would be less inclined to migrate if local promotion and professional development opportunities improved, underscoring the importance of retention in addressing workforce deficits (Pan American Health Organization 2023).

Limited, older Caribbean‐specific evidence reveals poor working conditions. A study across four English‐speaking Eastern Caribbean countries found unfavorable practice environments, particularly on staffing adequacy, nurse participation in hospital affairs, and managerial support (Lansiquot et al. 2012). A broader 2018 survey of 602 health workers across 14 Caribbean countries confirmed these perspectives, with 75% rating their working conditions unsatisfactory and 82% deeming them of low standard (Pan American Health Organization 2019). These environmental factors drive migration, with emigrants citing limited career advancement, poor treatment, a lack of professional respect, and inadequate supplies and facilities as the primary reasons for leaving (Pan American Health Organization 2019). The COVID‐19 pandemic further exposed health system vulnerabilities, compounding workforce challenges by affecting healthcare workers' physical and mental health (Pan American Health Organization 2023). This evidence suggests that nurses in the Caribbean may be functioning in an environment marked by inadequate resources, staffing, and leadership support and a lack of opportunity to be actively involved in care and policy decisions, but recent data are lacking.

Health workforce challenges within each Caribbean country exist in unique conditions. For example, there is great variation in the availability of nurses across countries, with 4.3 nurses per 10 000 individuals present in Suriname to 81.7 nurses per 10 000 individuals in Martinique (Pan American Health Organization 2019). In addition, Caribbean countries reflect a range of economic conditions as countries represent high, upper middle income, and lower middle income countries, which have implications for the resources available to support the delivery of care (Phelan et al. 2022; The World Bank 2025).

The available evidence indicates poor nurse working conditions and environment in the Caribbean may be pervasive and complex, warranting an urgent need to identify the most pressing issues that Caribbean nurses are facing. Caribbean nurses' work conditions, however, remain under‐described. Policies relevant to the Caribbean context have not been reported. Therefore, this study addresses these two research gaps.

We examined the perspectives of Government Chief Nursing Officers (GCNOs), the highest‐ranking nurses in each country's health system who oversee nursing workforce policy and practice nationwide. GCNOs were selected because they provide a broad view of the region, making their insights valuable for informing policy development by PAHO and countries' health ministries. The purpose of this study was to describe GCNOs' perspectives on concerns, strides, and policies to support the improvement of work environments for nurses and their patients. Our main research question was “what are the views of GCNOs on work environment concerns and how are these being addressed?”

## 2 Materials and Methods

### 2.1 Research Design

A qualitative descriptive design was used to provide a simple account of participants' narrative information without applying preconceived categories or a predetermined framework. This design was selected to describe nurse work environments from GCNOs' perspectives without deep theoretical interpretation. We aimed to obtain an account of nurse work environment in everyday terms. These choices influence study conclusions and transferability by excluding greater detail that could have been obtained through other data collection methods. We consider the findings transferable to countries with similar circumstances, including high out‐migration and nurse shortages. The 20 countries were selected as members of the Caribbean Community (CARICOM), an organizational entity for economic and policy coordination that is under the purview of the PAHO Caribbean Subregional Program Coordination. Primary data were collected through surveys completed by chief nurses or their designees between September and December 2024. Two authors have qualitative methods expertise. The remaining authors are health services researchers who have authored a qualitative research paper. An inductive content analysis was appropriate to describe and summarize GCNO perspectives from open‐text responses. Our interpretation was informed by our perspective as health services researchers. The Standards for Reporting Qualitative Research (SRQR) (O'Brien et al. 2014) checklist was used to certify that all research elements were completed.

### 2.2 Sample and Setting

A purposive sample included all GCNOs from Caribbean countries and territories (hereafter referred to as countries) under the purview of PAHO. Twenty countries were identified with guidance from PAHO Regional Advisors, and all GCNOs or principal nursing officers (PNO) in these countries were included in the sample. GCNOs typically operate within the Ministries of Health and oversee nursing and midwifery practice regulatory policy, and in some cases, education policy. The setting was the Caribbean health sector. The 20 countries comprised: Anguilla, Antigua and Barbuda, Bahamas, Barbados, Belize, Bermuda, British Virgin Islands, Cayman Islands, Dominica, Grenada, Guyana, Haiti, Jamaica, Montserrat, Saint Kitts and Nevis, Saint Lucia, Saint Vincent & the Grenadines, Suriname, Trinidad and Tobago, and Turks and Caicos.

### 2.3 Data Collection

Data were collected through collaboration among the WHO/PAHO Collaborating Centers (CCs) for nursing and midwifery (World Health Organization, n.d.). The group coordinated a joint survey to streamline Caribbean activities. A survey was prepared by all seven CCs in this group to encompass all relevant questions supporting each CC's designated activities in the Caribbean under their agreement with the WHO/PAHO. This comprehensive survey covered topics of advanced nursing practice, nursing education, and nursing care delivery. The data in this study comprises responses to three questions in the nursing care delivery component: (1) What are the major concerns regarding the work environment for nurses?, (2) What strides have been made in improving the work environment for nurses and their patients?, and (3) What policies have been implemented to support positive work environments for nurses and their patients?

The survey was promoted at a semi‐annual regional meeting of Caribbean chief nurses and distributed through formal communication from the PAHO Sub‐Regional Advisor for Human Resources for Health. PAHO and the two Caribbean CCs conducted additional outreach to increase response rates. The survey was hosted on a secure Qualtrics platform. Data collection occurred from September to December 2024. The data were managed on a secure password protected research server and compiled in an Excel file.

### 2.4 Ethical Considerations

The Pan American Health Organization Ethical Review Committee approved the initiative and determined it was not human subjects research; therefore, neither oral nor written consent was obtained. The survey was confidential, but not anonymous, as once a country is known, the GCNO can be identified. Data privacy was maintained using country pseudonyms. Respondents were informed their responses would be confidential with no identifiable data reported.

### 2.5 Data Analysis

Inductive content analysis was used to summarize the participants' free‐text responses to the three survey questions (Miles et al. 2014). All team members read the responses multiple times and met to discuss their overall sense of the data. Responses were divided into data units and assigned codes (i.e., short phrases that captured the essence of the text units). Because the responses were succinct and straightforward, agreement was easily reached for all codes.

Five broad topics were identified: physical environment, safety and security, staffing and workforce, professional development, and nurse well‐being. Five content analytic summary tables, one per topic, were developed to organize codes (Miles et al. 2014). The tables had a row for each participant and columns for concerns, strides, and policies implemented to support nurses' work environments.

A consensus‐based process was then used to group similar codes within each column into categories. One team member wrote a narrative description of the categories for each topic with verbatim quotes as supporting evidence. The descriptions were reviewed, modified through team discussion, and compiled to provide a summary of the participants' perspectives on nurses' work environments in the Caribbean.

To enhance trustworthiness, we applied the criteria of credibility, transferability, dependability, and confirmability (Korstjens and Moser 2018). Credibility was maintained through purposive sampling of national nursing authorities. Transferability was upheld by providing detailed de‐identified descriptions of the participants' responses and the research process so that this research can be independently evaluated. Dependability was achieved by providing descriptive quotes that support the study themes. Confirmability was achieved by using multiple coders and comparing their memos for a balanced and thorough analysis process.

## 3 Results

Nineteen of 20 GCNOs (95%) responded to these survey items. In two countries, two Health Ministry nursing officials responded jointly. In one country, in which the GCNO answered and invited the nursing association president to respond, both were included in the analysis because the GCNO responded about school nursing only and the association president responded about work environments broadly.

Five broad topics were derived from inductive content analysis as detailed in the data analysis section. These five topics reflected participants' concerns about the work environment for nurses in their countries: (a) physical environment, (b) staffing and workload, (c) safety and security, (d) professional development, and (e) nurse well‐being. For each of these concerns, the participants identified strides they made for improving the work environment for nurses and policies that had been implemented to support positive work environments. Their remarks about strides and policies overlapped considerably and are thus discussed together in the summaries below. These have been compiled by concern and are displayed in Table 1. One divergent view was expressed by Country O that due to the poor economic condition in their country “no improvement is possible.”

**TABLE 1:** Strides and policies in development or enacted related to nurse work environments concerns as reported by government chief nursing officers.

Concerns	Strides and policies
Physical environment	
Facilities in disrepair (i.e., poor lighting and ventilation, mold)	Assess and advocate for facility improvement Advocate for the maintenance team
Facilities in need of renovations	Identify facilities for refurbishing and retrofitting Identify facilities to be made climate smart Infrastructure improvement planning Timely reporting of infrastructure breakdown
Facilities not fit for their purpose	Commission and build new hospitals
Inadequate resources (i.e., medical supplies and equipment)	Equipment and supply audit Purchase equipment
Lack of ergonomic equipment and technology	Infrastructure improvements related to nurse comfort in the workplace
Lack of equipment that prevent injury of nurses	Gap analysis
Lack of dedicated spaces for nurses (i.e., break rooms for rest, dormitories, closets, on‐call rooms, changing facilities)	Formally request workstations
Lack of dedicated spaces for school nurses	Creation of a dedicated nursing space in every school Design spaces to provide care to students
Staffing and workload	
Human resource shortages	Submission of staffing needs
Inadequate staffing	Increased recruitment of registered nurses to ensure the necessary nurse‐to‐patient ratio is maintained in accordance with international standards
Understaffing	Nursing Services Workforce Plan Recruitment of nurses internationally
Inadequate supervision of inexperienced nurses	Improve nurse‐to‐patient and nurse‐to‐student ratios Develop recruitment protocol that led to more competent recruits FTE, skilled mixed
High patient‐to‐nurse ratios	Advocate for appropriate nurse to patient ratio
Decrease in standards and quality of work	Staffing criteria for safe staffing
Heavy workloads	Ensuring that there is always a second midwife or NICU nurse available
Decreased nurse well‐being	Remuneration for overtime for improved coverage Sessional allowances for overtime Flexible working hours and schedules
Absenteeism and low nurse retention	Recruitment and retention policy
Safety and security	
Threats to nurses' physical and psychological safety	Heightened surveillance, for example, CCTV.
Exposure to potential dangers in working environment	Hazard prevention education Risk identification
Limited safety measures in place for healthcare staff	Health and Safety Policy Safe practice
Spillover of community‐based violence into healthcare facilities (i.e., gun violence, gang activities)	Improved metal detector screenings in A/E Departments Zero tolerance policy against violence in the hospital
Escalating crime rates on hospital premises	Increase in security presence
Professional development	
Lack of structured professional development activities and education (i.e., enhanced orientation programs for nurses)	Continuing Education Programs
Lack of leadership training for supervisory roles	Increased training of nurses Leadership training
Lack of encouragement of young workforce to engage in professional development opportunities	Advocate for career development Mentorship and Preceptorship Programs
Scope of practice barriers (i.e., lack of autonomy to work to full extent of training)	Nursing Scope and Standards of Practice Advanced practice nurse prescriptive rights Expanding the scope of work of nursing support personnel to perform basic nursing duties
Nurses' well‐being	
High stress levels and burnout	Promote good mental health Reduce stress and burnout Identification of emotional support staff Post event counseling
Poor work‐life balance	Encouraging a positive work‐life balance
Low job satisfaction	Enhance job satisfaction Grievance reporting Internal employee satisfaction surveys
Problems with teamwork, communication, and collaboration	Promote teamwork Code of Conduct
Practical barriers to nurses' work life (i.e., inadequate public transportation, lack of childcare)	Transportation improvements: alignment of schedule with public transit, provide a private bus service, reduce taxes for nurses' personal vehicles

### 3.1 Physical Environment

Eleven participants expressed concerns with the physical environment in which nurses worked. Several revealed healthcare facilities in their countries were in disrepair, in need of renovations, and not fit for their purpose. One mentioned poor lighting and ventilation as well as mold. Some revealed that the facilities had inadequate resources. One participant wrote, “The major concerns regarding work environment for nurses are limited resources. For example, medical supplies, and medical equipment” (Country M). Participants indicated they needed equipment and technology that was ergonomic and could prevent injuries to nurses. A few participants pointed out that there was a lack of dedicated spaces for nurses in their facilities, including break rooms for rest, dormitories, closets, on‐call rooms, and changing facilities. One participant pointed out that school nurses lacked dedicated space in schools.

Many participants shared strides and policies they, their institutions, and their countries had made to improve their facilities. Several participants were currently assessing and advocating for facility improvement. Some had identified facilities for refurbishing or climate renovations, completed equipment and supply audits, conducted gap analyses, advocated for a maintenance team, and requested workstations. One participant described an “audit of equipment and supplies which lead to the Ministry purchasing equipment” (Country H). Some participants described how their countries were retrofitting facilities, building new hospitals, and purchasing equipment and resources. One participant mentioned that schools had created a dedicated space for nurses to provide care in every school.

### 3.2 Staffing and Workload

Ten participants expressed concerns about staffing and workload, citing staff shortages, inadequate staffing, and understaffing. Some noted shortages of senior staff, resulting in inadequate supervision of inexperienced nurses. Several participants reported that staff shortages resulted in high patient‐to‐nurse ratios and a decrease in standards and quality of work. Others mentioned the consequences of staff shortages and heavy workloads on nurse well‐being, as well as increased absenteeism and challenges with retention. One participant expressed concerns about “long hours due to staffing shortages, increase in stress level, having to deal with difficult patients, sometimes poor patient outcomes” (Country J).

Participants also described strides and policies implemented to address staffing and workload issues. Several reported efforts to improve or advocate for better nurse‐to‐patient ratios. Others described recruitment strategies, including international recruitment, increasing recruitment of registered nurses to comply with international standards, developing a recruitment protocol that led to more competent recruits, and developing a new recruitment and retention policy. Some addressed staffing shortages through strategically scheduling the right “mix” of nurses, such as increasing nurse managers, ensuring availability of specialized skills (e.g., midwifery), and expanding support personnel roles. Several participants mentioned compensating nurses for overtime or providing flexible scheduling to ensure coverage. Some participants listed a number of strategies to address staffing and workload. One wrote, “Recruitment of nurses internationally to fill the gap, aim to promote good mental health, advocate for career development, finding ways of reducing stress and burnout in order to provide quality care, offer flexible work schedules where possible.” (Country J).

### 3.3 Safety and Security

Seven participants expressed safety and security concerns. Participants reported that limited safety measures left nurses feeling vulnerable to physical and psychological threats, creating precarious and stressful working conditions. One participant was particularly concerned about the safety of nurses working in accident and emergency departments and psychiatric facilities (Country L).

Participants also described how community‐based violence penetrated healthcare environments. They described how gang activities and gun violence would penetrate healthcare facilities. One participant described “spillover community gun violence into clinical settings (e.g., offenders entering clinical settings to ensure that gunshot victims have been killed)” (Country A). The participants suggested that crime rates on hospital premises were escalating and exacerbated workplace safety concerns.

Participants shared strides and policy initiatives they and their institutions implemented to address the safety and security of healthcare personnel. Some reported educational programs focused on hazard prevention were developed and were well received by nurses who felt more prepared to manage threats of violence. Several participants reported that their institutions had increased the presence of security and police officers to address external threats of violence and potential safety breaches. Others noted their institutions had increased security measures, including enhanced metal detector screenings in accident and emergency departments, increased CCTV surveillance, and automated entrance and exit doors. One participant welcomed a “police officer stationed at the accident and emergency department” and “security officers employed and assigned to the wards at the psychiatric hospital” (Country L). Participants also reported the implementation of a zero‐tolerance policy against workplace violence in their institutions. They identified the need for proactive risk identification and ongoing development of health and safety policies to create a secure workplace. One participant identified the importance of a “zero tolerance policy against violence in the hospital” (Country F).

### 3.4 Professional Development

Five participants described professional development challenges related to education and training and scope‐of‐practice limitations. Some mentioned the lack of structured professional development, including enhanced orientation programs and leadership training for supervisory roles. One participant shared the challenge of having a young workforce that is not encouraged to engage with professional development opportunities. Several mentioned the need to provide opportunities for the development of nurse leaders. Others mentioned nurses' professional development was hindered by scope‐of‐practice barriers, with one noting a “lack of autonomy to work to full extent of training.” (Country H).

Participants reported strides and policy initiatives to address these challenges, advocacy for additional training and continuing education for nurses in areas such as humanized care, social services, leadership, mentorship, and precepting. One participant shared details of their continuing education strategy, “Continuing Education Programs: encourage and support nurses in attending continuing education programs to keep their knowledge and skills current and enable them to grow in their careers. Mentorship and preceptorship programs: Pair experienced nurses with less experienced colleagues for mentoring and support, contributing to professional development and improved patient care” (Country O). Related to scope‐of‐practice barriers, one respondent noted expanding roles for nursing support personnel and allowing advanced practice nurses prescriptive authority. Some participants supported leadership development by providing protected time for leadership activities as well as assessing the needs of nursing leaders. One participant wrote, “Allocate time for the leadership and management of nursing staff, which encompasses performance appraisals, performance reviews, supervision, support, and the education of new staff members” (Country R).

### 3.5 Nurses' Well‐Being

Four participants expressed concerns about nurses' well‐being, noting high levels of stress and burnout among nurses. Threats to well‐being could be related to poor work‐life balance, low job satisfaction, and problems with teamwork, communication, and collaboration. Several participants also identified fear of violence as a significant stressor. Some noted that stressors stemmed from practical barriers, such as transportation and childcare. One participant reported difficulties related to “transportation, particularly on holidays and weekends when public transit options are not available.” (Country A).

Participants shared strides and policies to address nurse well‐being, including efforts to encourage work‐life balance, mental health, and job satisfaction, and to reduce burnout. Others addressed nurses' well‐being by developing a code of conduct. The participants also indicated that they provide emotional support for staff who experienced emotional distress, such as post‐event counseling following traumatic events. Several suggested that improving communication between nurses and administrators, such as by employing “internal employee satisfaction surveys” and instituting “grievance redress mechanism[s]” (Country K), could improve nurses' morale. They also mentioned practical solutions to transportation challenges including aligning nursing schedules to public transportation schedules, using private chartered buses to transport nurses to work, and reducing taxed on nurses' personal vehicles.

## 4 Discussion

This paper provided the perspectives of 19 Caribbean GCNOs on concerns, strides, and policies related to nurse work environments. Utilizing inductive content analysis, GCNOs identified concerns on the physical environment; staffing and workload; safety and security; professional development; and nurse well‐being. Findings contribute to the development of human resources policy in this region to address ongoing migration in the context of poor working conditions.

GCNOs reported various policy responses, including procedures, surveys, regulatory changes, strategic planning, and legislative amendments. These demonstrate varying degrees of implementation across countries. Most policies focused on health and safety, with additional attention to staffing, professional development, workplace standards, and organizational policies.

Our findings align and extend recent global, regional, and scholarly literature regarding nursing work environments, including working conditions, safety, and violence prevention. Across the literature, working conditions and workplace safety consistently emerge as contributing factors to well‐being, retention, and system performance. Our findings align with these greater patterns while bringing to light context‐specific challenges for Caribbean nursing leaders. This synthesis provides a foundation for considering the policy and leadership implications of Caribbean region nursing work environments.

While most countries report regulations addressing nurses' working conditions, gaps remain in workplace safety and violence prevention. State of the World's Nursing reports (World Health Organization 2020, 2025b) illustrate that preventive measures against attacks are less consistently implemented than regulations regarding hours, conditions, and general safety. The Americas, including the Caribbean, lag global averages. Our findings reflect this: Caribbean GCNOs were concerned about safety and security and simultaneously described efforts to strengthen protective measures. Formal regulatory frameworks are insufficient to ensure safe working environments, and critical determinants of effectiveness include enforcement, resourcing, and leadership engagement.

Global surveys, regional policy analysis, and GCNOs consistently document workplace safety and violence concerns. Survey data from national nursing association presidents indicates that exposure to workplace violence remains widespread and that existing prevention policies are only partially effective (Sharplin et al. 2025). Regional policy analyses document ongoing efforts to strengthen occupational health and safety through enhanced security, infection prevention, and zero‐tolerance workplace violence policies (Pan American Health Organization 2024). This literature aligns with our findings among CGNOs who frequently identify safety and security as both a pressing concern and an area of active intervention. Notably, CGNOs relay context‐specific threats, such as gun violence, alongside organizational responses including increased surveillance and security personnel. This underscores the need for enforceable strategies rather than reliance on generic policies alone.

Although the Caribbean nations in this study range from lower‐middle‐income to high‐income, their healthcare systems have structural traits that are frequently found in low‐ and middle‐income countries (LMIC). Concerns about the physical workplace, staffing shortages, and resource limitations are common in all settings, but are especially noticeable in areas with underdeveloped health system infrastructure (Rani and Bashir 2024). These trends are closely reflected in the issues raised by Caribbean GCNOs, with the physical environment being the most mentioned difficulty. This convergence suggests that national income classification may obscure underlying health system fragilities and that economic growth has not consistently translated into improved working conditions for nurses within the Caribbean healthcare sector.

Migration literature underscores the central role of working conditions in shaping nurse retention and mobility, particularly in settings characterized by resource constraints. Both systematic and integrative reviews of nurse and health workers consistently identify poor working conditions, heavy workloads, inadequate staffing, and limited access to resources as drivers of emigration from source countries (Konlan et al. 2023; Toyin‐Thomas et al. 2023). The findings mirror the concerns expressed by Caribbean GCNOs, who highlight challenges related to physical infrastructure, workforce shortages, and insufficient institutional support for recruitment and retention. This alignment suggests that suboptimal working conditions in the Caribbean may serve as push factors like those in LMIC contexts. Workplace‐focused strategies are important in efforts to stabilize the nursing workforce.

Our findings align with global health workforce goals, such as Sustainable Development Goal #8.8 set by the World Health Organization: “protect labor rights and promote safe working environments” (World Health Organization 2022) and the 2025 State of the World's Nursing, which delineates a strategic direction for service delivery: “Nurses work to the full extent of their education and training in safe and supportive work environments.” (World Health Organization 2025b, 65). Accordingly, our findings illustrate that the lack of safe and supportive working environments is a pressing current concern in the Caribbean region.

Our findings likewise align with recent developments in health workforce policy, particularly nurse migration trends. In May 2025, the World Health Assembly (WHA) extended the 2021–2025 Global Strategic Directions for Nursing and Midwifery to 2030, which include for service delivery, “work to the full extent of their education and training in safe and supportive service delivery environments, and for leadership, ‘invest in leadership skills development’” (World Health Organization 2021). The WHA also adopted Resolution WHA78.16, urging member states to “promote decent work, formation recognition, fair and equal remuneration, supportive working environments and equal access to education, training, employment and career progression opportunities” (World Health Organization 2025a). These recent policy developments complement the concerns expressed by the GCNOs and our findings illustrate a range of member state actions to fulfill these priorities.

In the context of prior systematic or integrative reviews, our study's unique contribution is the focus on the Caribbean and the perspective of GCNOs. Participants from 19 of 20 countries under PAHO's jurisdiction provided a definitive description of the priority concerns of nurse authorities about nurses' work conditions in this region. Based on the literature, our findings seem generalizable to countries in similar nurse shortage and migration pressures.

Our findings prompt new research questions including exploration of policy effectiveness and the implementation barriers in specific countries. Implementation barriers might reveal why some countries are slower to adopt certain policies (e.g., staffing ratios, violence prevention) perhaps due to limited resources or political instability. Another research priority to inform policymakers in the region would be to learn the policies' long‐term impact. Soliciting the perspectives of front‐line nurses and nurse managers would add important insights and may validate these findings.

### 4.1 Study Limitations

The survey's broad questions allowed contextual interpretations, but limited in‐depth probing, restricting findings to overarching concerns and policy responses. Lack of probing might have impacted the depth of insights, for example, illuminating policy effectiveness. While open‐ended questions were appropriate for exploratory work, transferability may be limited by varying GCNO roles across countries. Limiting the sample to GCNOs introduces potential bias, as respondents report on systems they lead, creating incentives to emphasize achievements over shortcomings. Their system‐level perspective may also be insulated from frontline realities, particularly in under‐resourced settings, overstating policy progress relative to nurses' lived experiences. While we value frontline perspectives, GCNOs were selected for their broad understanding of regional workplace concerns and policy responses. Despite these limitations, findings are considered trustworthy due to rigorous analytic processes.

## 5 Conclusion

The concerns and associated efforts reflect a global commitment to enhancing nursing environments through targeted strategies addressing physical environment staffing, safety, professional development, and nurse well‐being. While improving nurse working conditions is expected to enhance patient care, policy implementation varies significantly across countries. Recommendations from this study are relevant for Caribbean governments', institutional managers, and professional nursing associations. For regions facing similar challenges, our findings offer policy alternatives and show that GCNOs have an important role in policy development.

## 6 Relevance to Nursing and Health Policy

These results have significant ramifications for Caribbean leadership and nurse workforce policy. First, formal regulations do not ensure secure or encouraging workplaces. Sustained enforcement, adequate resourcing and leadership authority are necessary to translate policy into practice. Second, workplace safety, especially violence prevention, remain critical and unresolved workforce issues that require coordinated efforts among regulators, employers and leadership instead of isolated institutional responses. Third, persistent deficiencies in the work environment and staffing conditions suggest that workforce challenges cannot solely be resolved through recruitment. Systemic investment in infrastructure and working conditions will be required. Increasing GCNOs' abilities to promote workplace safety programs, influence policy implementation, and match retention tactics with more general health system objectives may lessen migration pressures and increase worker stability. When taken as a whole, these implications highlight the necessity of workforce strategies that are context‐sensitive, enforced, and prioritize nurse safety and working conditions as fundamental components of health system performance.

Our findings have implications for health policy in the Caribbean and countries facing similar challenges. Our results show that challenges in providing decent working conditions are being addressed differently across the region. For example, in patient and staff health and safety, we identified 10 policies across six countries. Of these six countries, one country reported four policies while four reported just one policy. Given the wealth of policy effort, an important next step would be for GCNOs to share among themselves their policy efforts on nurse work environments. This sharing could be a routine agenda item in the CARICOM biannual meetings. Given their firsthand knowledge, GCNOs should be included in health ministers' decision‐making processes for policy development and implementation. While the recommendations are regionally relevant, we note that given diversity in the region, some countries may face specific challenges that require customized solutions.

It is tempting to select initiatives that are operating sporadically across the region and that could benefit the nursing workforce if more widely promoted and implemented. Given the variation of the priority concerns and local context of countries, however, one or two solutions are not identifiable. Each country should begin with their priority concern recognizing that all concerns shared by the GNCOs are interrelated in their effect on working conditions and migration. As the GCNOs reported 52 different policy actions ranging from very specific training, regulation, and infrastructure development to overarching nurse job outcome goals, implementation guidance will necessarily be tailored to specific policy actions.

As the institutional manager holds the crucial role in improving nurses' working conditions, our findings imply a need for governmental and managerial collaboration. To inform this collaboration, managers should consult internally and share findings externally. Internally, they should communicate openly with the staff to learn about and respond to work environment concerns in real time, conduct periodic evaluation of work conditions and policy effectiveness. Externally, they could prepare and provide reports of their evaluation findings to support requests for budgetary allocations and resources from regional ministry authorities.

The concerns about nurses' professional development may be addressed through programs developed and disseminated by the professional nursing association, the Caribbean Nurses Organization. The research implication of our work is to replicate this survey after 5 years to evaluate progress from the chief nurses' perspective.

## Author Contributions

**Eileen T. Lake:** conceptualization, investigation, writing – original draft, methodology, validation, writing – review and editing, project administration, data curation, supervision, resources. **Domenique Villani:** conceptualization, investigation, writing – original draft, methodology, validation, writing – review and editing, formal analysis. **Lynne Moronski:** conceptualization, investigation, writing – original draft, methodology, validation, writing – review and editing, formal analysis. **Lindsey Lee:** conceptualization, investigation, writing – original draft, methodology, validation, writing – review and editing. **Sherif Olanrewaju:** conceptualization, investigation, writing – original draft, methodology, validation, writing – review and editing, formal analysis. **Norah Solaiman:** conceptualization, methodology, writing – review and editing. **Claire Burke Draucker:** conceptualization, investigation, writing – original draft, methodology, validation, writing – review and editing, formal analysis.

## Funding

Research reported in this publication was supported by the National Institute of Nursing Research of the National Institutes of Health under Award Number T32NR007104. The content is solely the responsibility of the authors and does not necessarily represent the official views of the National Institutes of Health.

## Ethics Statement

Ethical approval was granted by the Pan American Health Organization Ethical Review Committee Ref. No: PAHOERC.0733.01.

## Conflicts of Interest

The authors declare no conflicts of interest.

## Data Availability

The data that support the findings of this study are available from the corresponding author upon reasonable request.
